# Authentication of *Ginkgo biloba* Herbal Products by a Novel Quantitative Real-Time PCR Approach

**DOI:** 10.3390/foods9091233

**Published:** 2020-09-04

**Authors:** Liliana Grazina, Joana S. Amaral, Joana Costa, Isabel Mafra

**Affiliations:** 1 REQUIMTE-LAQV, Faculdade de Farmácia, Universidade do Porto, Rua de Jorge Viterbo Ferreira, 228, 4050-313 Porto, Portugal; li_grazina@hotmail.com (L.G.); jbcosta@ff.up.pt (J.C.); 2 Centro de Investigação de Montanha (CIMO), Instituto Politécnico de Bragança, Campus de Sta. Apolónia, 5301-857 Bragança, Portugal; jamaral@ipb.pt

**Keywords:** adulteration, authenticity, *Ginkgo biloba*, plant infusions, real-time polymerase chain reaction

## Abstract

*Ginkgo biloba* is a widely used medicinal plant. Due to its potential therapeutic effects, it is an ingredient in several herbal products, such as plant infusions and plant food supplements (PFS). Currently, ginkgo is one of the most popular botanicals used in PFS. Due to their popularity and high cost, ginkgo-containing products are prone to be fraudulently substituted by other plant species. Therefore, this work aimed at developing a method for *G. biloba* detection and quantification. A new internal transcribe spacer (ITS) marker was identified, allowing the development of a ginkgo-specific real-time polymerase chain reaction (PCR) assay targeting the ITS region, with high specificity and sensitivity, down to 0.02 pg of DNA. Additionally, a normalized real-time PCR approach using the delta cycle quantification (ΔCq) method was proposed for the effective quantification of ginkgo in plant mixtures. The method exhibited high performance parameters, namely PCR efficiency, coefficient of correlation and covered dynamic range (50–0.01%), achieving limits of detection and quantification of 0.01% (*w*/*w*) of ginkgo in tea plant (*Camellia sinensis*). The quantitative approach was successfully validated with blind mixtures and further applied to commercial ginkgo-containing herbal infusions. The estimated ginkgo contents of plant mixture samples suggest adulterations due to reduction or almost elimination of ginkgo. In this work, useful and robust tools were proposed to detect/quantify ginkgo in herbal products, which suggests the need for a more effective and stricter control of such products.

## 1. Introduction

Ginkgo (*Ginkgo biloba* L.) is a millenary Chinese tree that belongs to the Ginkgoaceae family whose leaves are widely used for medicinal purposes [1]. Owing to its composition in pharmacologically active compounds, such as flavonol glycosides and terpene trilactones (bilobalides and ginkgolides) [2,3], ginkgo is used for its capacity to improve cognitive impairment in the elderly and quality of life in mild dementia. It is also known for its therapeutic action in peripheral circulatory illnesses, improving blood circulation and preventing clot formation [1,2,4,5,6]. Currently, different herbal products that have ginkgo as an ingredient are readily available in the global market, including in plant food supplements (PFS) and herbal infusions. According to recent surveys, ginkgo was the most popular botanical in PFS and is used in six European Union countries [7], while in the United States it ranked among the top 10 dietary supplements in the category of herbal/botanicals [8]. Moreover, the global market of *G. biloba* extracts, mainly intended for pharmaceutical and food supplement industries, was estimated to be US $1590.5 million in 2018 and projected to reach US $2379.2 million by 2028 [9]. The high demand of ginkgo in the global market and the increased value of ginkgo products make them potential targets for economically motivated adulteration. Frauds can be performed by the total or partial replacement of ginkgo with other plant species or by adding pure flavonols/flavonol glycosides or extracts (rich in flavonol glycosides) from other plant species, such as *Styphnolobium* japonicum (syn: *Sophora japonica*) and *Fagopyrum esculentum* Moench, belonging to the Fabaceae and Polygonaceae families, respectively [6].

Both pharmaceuticals and traditional herbal medicinal products (THMP) (either final products or the extracts used for their production) must comply with the Pharmacopeia standards, established for ginkgo leaves or extracts, to ensure the product’s quality [3,10]. However, in the case of other ginkgo-containing products, such as herbal infusions and PFS that are legally considered as foods, they do not have to comply with those standards. Moreover, in these type of products, previous studies have reported adulterations associated with the partial or complete replacement of ginkgo with other plants [1,3]. Thus, it is crucial to provide analytical tools that allow the identification and quantification of *G. biloba* in herbal products classified as foods, making possible the verification of compliance with label statements.

Several analytical methodologies have been proposed for authenticity assessment of ginkgo-containing herbal products based on liquid chromatography coupled to mass spectrometry (LC-MS), high performance thin layer chromatography (HPTLC), HPTLC coupled with nuclear magnetic resonance and spectroscopy [1,3,11,12,13,14]. Those methodologies rely on the identification of bioactive compounds and/or chemical profile, which can be affected by several external factors, such as the plant part/tissue, plant age, environmental conditions, geographical location, and storage conditions, among others. Furthermore, chemical approaches can be less adequate when the formulation includes several plant species. On the contrary, DNA-based methodologies have been shown to be suitable tools for the identification/discrimination of species due to their high specificity and sensitivity, with different works reporting successful applications in the authentication of herbal products, namely food supplements or herbal infusions [15,16,17]. In this regard, different approaches including species-specific polymerase chain reaction (PCR), multiplex PCR, real-time PCR, high resolution melting (HRM) analysis, sequence characterization of amplified regions (SCAR), DNA barcoding, and next generation sequencing (NGS), among others, have been proposed to authenticate medicinal plants in herbal products [18]. Among them, real-time PCR offers the advantage of providing quantitative information, being a very sensitive, specific, and fast tool.

So far, only a few works regard the identification of *G. biloba* in herbal products and PFS using DNA-based approaches. Little [19] proposed the use of DNA barcoding targeting a short region of *matK* gene to identify gingko in PFS. Despite using a DNA mini-barcode (166 bp), 3 out of 40 samples were not successfully amplified. Besides, it should be noticed that this approach is not adequate for samples containing mixtures of ingredients/medicinal plants. Liu et al. [20] developed a rapid identification method to detect both gingko and a possible adulterant (*Sophora japonica*) in herbal products using a recombinase polymerase amplification (RPA) approach, which relied on the use of species-specific primers and a probe with high specificity, though with limited cross-reactivity testing. More recently, Dhivya et al. [21] developed a real-time PCR assay using a species-specific hydrolysis probe to identify *G. biloba* in natural health products. The method allowed the specific and sensitive detection of *G. biloba*, but without any quantitative analysis that should rely on the development of an adequate calibration model. Besides, the authors did not demonstrate its applicability in the analysis of processed/complex products. Therefore, the present work aimed at filling this gap by providing a specific, sensitive, high-throughput and cost-effective real-time PCR method that, besides establishing the unequivocal identification of *G. biloba* in herbal products, enables its quantification in plant mixtures. For this purpose, a normalized quantitative method was proposed, which was further validated and applied to assess the authenticity of ginkgo-containing commercial herbal infusions and to verify their labelling compliance.

## 2. Materials and Methods

### 2.1. Plant Species and Commercial Samples

Leaves from *G. biloba* were kindly provided by the Botanical Garden of University of Porto, Botanical Garden of Bern, Serralves Garden and Botanical Garden of Madeira (Table S1, Supplementary Material). Leaves or seeds of 73 plant species corresponding to medicinal plants, fruits and spices were used for cross-reactivity testing (Table S1, Supplementary Material). A total of 20 herbal infusions were bought at local stores, including specialized herbalists, and from the internet (Table 1).

For method development, model mixtures with known amounts of dried leaves of *G. biloba* in *Camellia sinensis* were prepared to contain 50%, 10%, 5%, 1%, 0.5%, 0.1%, 0.05% and 0.01% (*w*/*w*). Firstly, a reference mixture with 50% of *G. biloba* was prepared by adding 10 g of ground *G. biloba* leaves to 10 g of ground plant material of *C. sinensis*. All the subsequent mixtures were prepared by sequential additions of *C. sinensis* plant material up to the level of 0.01% (*w*/*w*). For method validation, blind mixtures were independently prepared as described for the reference mixtures, with the proportion of 20%, 8%, 2%, and 0.2% (*w*/*w*) of *G. biloba* in *C. sinensis* plant material and were further analyzed as unknown samples.

Seeds were ground with a mortar, while the leaves and herbal infusions were ground in a laboratory mill Grindomix GM200 (Retsch, Haan, Germany).

### 2.2. DNA Extraction

The NucleoSpin Plant II kit (Macherey-Nagel, Düren, Germany) was chosen to perform the DNA extraction from 50 mg of each sample, according to the manufacturer’s instructions with slight modifications, as described by Costa et al. [22]

### 2.3. DNA Quality and Purity

The yield and purity of DNA extracts were assessed by UV spectrophotometry using a Take3 micro-volume plate accessory, on a Synergy HT multi-mode microplate reader (BioTek Instruments, Inc., Winooski, VT, USA). The nucleic acid protocol was set for double-strand DNA in the Gen5 data analysis software version 2.01 (BioTek Instruments, Inc., Winooski, VT, USA), which was applied to absorbance data measured at 260 and 280 nm.

The quality of DNA extracts was further assessed by electrophoresis with 1% of agarose gel as previously described [22].

### 2.4. Target Gene Selection, Oligonucleotide Primers and Probes

A set of primers (Gkb2-F/Gkb2-R) and a specific probe (Gkb2-P) labelled with fluorescein (FAM) as a fluorescent reporter and black hole quencher 1 BHQ-1 as quencher, were designed to target the Internal Transcribed Space (ITS) region of *G. biloba* (GenBank: Y16892.1) (Table 2). In silico analysis of sequences and primers was performed using the BLAST and Primer-BLAST tools to verify fragment and primer specificity, respectively. OligoCalc software was used to check primer properties and ensure the absence of primer hairpins and self-hybridization.

To ensure the presence of amplifiable DNA, a universal eukaryotic primer pair (EG-F/EG-R), targeting a conserved 18S rRNA nuclear region, was used [23]. The same primer pair together with a probe (EG-P) was used as an endogenous control gene for developing the normalized real-time PCR system [24]. The primers and probes were synthesized by Eurofins MWG Operon (Ebersberg, Germany).

### 2.5. Qualitative PCR

PCR amplification was performed using a total reaction volume of 25 μL which contained 20 ng of DNA, buffer (67 mM Tris-HCl, pH 8.8, 16 mM (NH_4_)2SO_4_, 0.01% Tween 20), 3 mM of MgCl_2_, 1.0 U of SuperHot Taq DNA Polymerase (Genaxxon Bioscience GmbH, Ulm, Germany), 280 nM of each primer and 200 μM of dNTP (Grisp, Porto, Portugal) (Table 2). The reactions were carried out in a MJ Mini™ Gradient Thermal Cycler (Bio-Rad Laboratories, Hercules, CA, USA), using the following optimized programs: initial denaturation at 95 °C for 5 min; 35 or 40 cycles (for EG-F/EG-R or Gkb2-F/Gkb2-R primers, respectively) of amplification at 95 °C for 30 s, 63 °C or 62 °C (for EG-F/EG-R or Gkb2-F/Gkb2-R primers, respectively) for 30 s and extension at 72 °C for 30 s; and a final extension at 72 °C for 5 min.

PCR products were further analyzed by electrophoresis in a 1.5% agarose gel stained with 1× Gel Red (Biotium, Hayward, CA, USA) and running in 1× SGTB buffer (GRISP, Porto, Portugal) for 20–25 min at 200 V. Each extract was amplified in at least two independent assays.

### 2.6. Real-Time PCR

The reactions were performed using 20 μL of total reaction volume, containing 2 µL of DNA (20 ng), 1× SsoFast Probes Supermix (Bio-Rad Laboratories, Hercules, CA, USA), 300 nM or 400 nM of each primer set (EG-F/EG-R or Gkb2-F/Gkb2-R, respectively) and 200 nM of each probe (EG-P or Gkb2-P, for eukaryotic and *G. biloba* genes, respectively). A fluorometric thermal cycler CFX96 Real-time PCR Detection System (Bio-Rad Laboratories, Hercules, CA, USA) was used to amplify, simultaneously and in parallel reactions, each target sequence, under the following conditions: 95 °C for 5 min, 45 cycles at 95 °C for 15 s and 65 °C for 45 s, and the fluorescence signal was collected at the end of each cycle. The data evaluation, from each real-time PCR assay, was made using the software Bio-Rad CFX Manager 3.1 (Bio-Rad Laboratories, Hercules, CA, USA). Real-time PCR assays were performed, at least, in two independent runs using *n* = 3 or *n* = 4 replicates in each one.

For the construction of a calibration curve and for the determination of the absolute limits of detection (LOD) and quantification (LOQ), 10-fold serially diluted ginkgo DNA extracts (20 ng–0.002 pg) were amplified by real-time PCR. Additionally, a normalized calibration model was constructed based on the parallel amplification of the ITS1 region of *G. biloba* (target sequence) and the 18S rRNA gene (reference for eukaryotes) using the model mixtures (0.01–50%) of *G. biloba* in *C. sinensis*. The acceptance criteria established for real-time PCR assays were the PCR efficiency between 90–110%, the slope within −3.6 and −3.1 and the correlation coefficient (*R^2^*) above 0.98 [25,26]. The lowest amplified level for 95% of the replicates was considered as the LOD and the LOQ was set as the lowest amplified level within the linear dynamic range of the calibration curve, which should cover a minimum of 4 orders of magnitude and should extend to ideally 5 or 6 log_10_ concentrations [25,26].

## 3. Results and Discussion

### 3.1. DNA Quality and Selection of Target Region

In general, DNA extracts from the leaves, seeds and commercial samples showed adequate yields and purities, being in the range of 17.6–270.8 ng/µL and 1.4–2.1, respectively. Before the *G. biloba* specific amplification of target region, all extracts were tested by PCR targeting a universal eukaryotic region (EG-F/EG-R) to check the capacity of DNA amplification and avoid false negatives [23]. All DNA extracts used for reactivity testing were amplified (Table S1, Supplementary Material).

So far, different regions have been assessed, either as a single locus or in combination, for their adequacy as barcode markers in plant species, which include *matk*, *rbcL*, ITS and ITS2, among others [27]. In this work, the non-coding ITS region of nuclear ribosomal DNA was selected due to its high power of species discrimination over plastid regions, allowing the differentiation of closely related species [27,28,29]. This region has been previously proposed for the development of PCR assays using species-specific primers aiming at identifying medicinal plant species, with high specificity and sensitivity [30,31]. The specificity of the newly designed primers (Gkb2-F/Gkb2-R) was initially in silico verified and subsequently assayed experimentally against different DNA extracts from several plant species (*n* = 73). As expected, the primers proved to be specific since only the DNA extracts from *G. biloba* were amplified (Table S1, Supplementary Material). Afterwards, the optimized species-specific PCR assay, using a 10-fold serially diluted *G. biloba* DNA extract (20 ng), showed a sensitivity down to 0.002 ng (Figure S1, Supplementary Material) and was further applied in the analysis of the commercial samples (Table 1). The achieved sensitivity was much higher than that obtained by the RPA-lateral flow strip device reported by Liu et al., which was approximately 1 ng of purified DNA. Moreover, only a few plant species were used for cross-reactivity testing (*Crataegus pinnatifida*, *Epimedium brevicornu*, *Selaginella tamariscina* and *Arisaema heterophyllum*) by those authors. In the same work, a species-specific PCR assay targeting *G. biloba* DNA was also developed, but again with very limited specificity testing (only against *S. japonica*).

### 3.2. Quantitative Real-Time PCR

#### 3.2.1. Method Development

**Figure 1:**
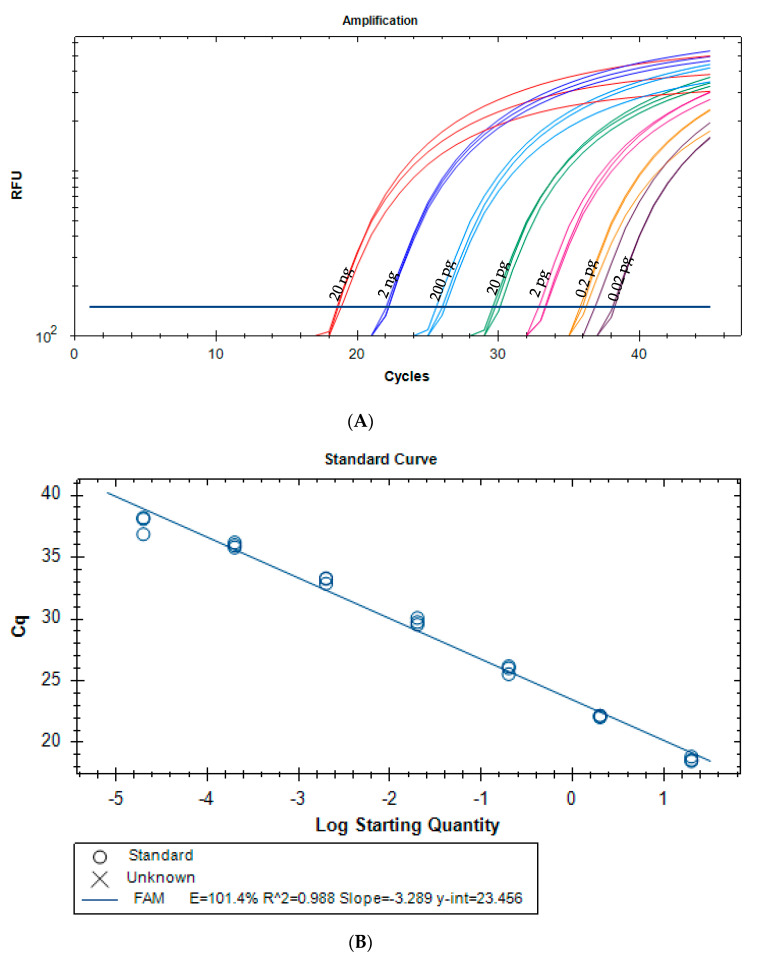
Amplification curves (**A**) and respective calibration curve (**B**) of a real-time PCR assay with a hydrolysis probe targeting ITS1 region of *G. biloba*. The amplified extracts correspond to 10-fold serially diluted ginkgo DNA from 20 ng to 0.002 pg (*n* = 3 replicates). Cq (cycle of quantification, also known as Ct, cycle threshold).

Following the demonstrated suitability of the proposed primers for *G. biloba* specific detection, a real-time PCR method was developed using a newly designed hydrolysis probe (Gkb2-P), increasing the sensitivity and specificity of the assay. Figure 1 presents the real-time PCR amplification curves and respective calibration curve using a 10-fold serially diluted ginkgo DNA extract. The average parameters of PCR efficiency (101.4%), slope (−3.284) and *R*^2^ (0.988) were all within the acceptance criteria (Figure 1B), suggesting a high performance of the assay [25,26]. The dynamic range covered six orders of magnitude of the target analyte (20 ng to 0.02 pg of ginkgo DNA) and the absolute LOD of the real-time PCR assay was established as 0.02 pg of *G. biloba* DNA, corresponding to 0.285 genomic DNA copies (using the mean value of the Plant DNA C-value database [32]) and considering the amplification of all replicates (*n =* 6 from two independent assays). Since the LOD value was within the linear dynamic range of the calibration curve, the LOQ value was set at the same value (0.02 pg) [25,26].

**Figure 2:**
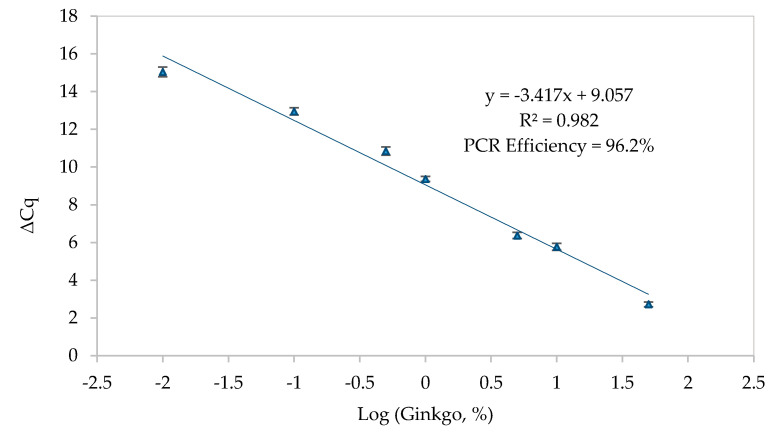
Normalized calibration curves obtained by real-time PCR, targeting the ITS1 region of ginkgo, using the binary mixtures of *G. biloba* in *C. sinensis* (50%, 10%, 5%, 1%, 0.5%, 0.1%, 0.05% and 0.01% (*w*/*w*)). The normalized ΔCq method was performed by the parallel amplification of a eukaryotic sequence (18S rRNA) as reference (mean values of six independent assays with *n* = 3 replicates).

For establishing a quantitative model of ginkgo in herbal material, a normalized real-time PCR assay using the ∆Ct method was developed. This approach accounts with amplification variations due to inconsistent DNA recovery and quality/degradation among extracts as a result of processing [24,33,34,35]. It relies on the construction of a normalized calibration curve using the cycle of quantitation (Cq) values from the target region (ITS1) and a reference endogenous gene (nuclear 18S rRNA) by applying the expression ΔCq = Cq (ginkgo)−Cq (universal gene). The normalized calibration curve was obtained by plotting the calculated ΔCq values versus the logarithm of the gingko concentration, using the binary mixtures with known quantities of *G. biloba* in *C. sinensis* (50.0%, 10.0%, 5.0%, 1.0%, 0.5%, 0.1%, 0.05%, and 0.01%, *w*/*w*) (Figure 2). The choice of *C. sinensis*, also commonly known as the “tea plant”, to prepare the reference mixtures was based on the high frequency of its use in mixed herbal infusions. The developed normalized real-time PCR approach exhibited high performance, as inferred from the obtained parameters of PCR efficiency (96.2%), *R^2^* (0.982) and slope (−3.417) (mean values from 6 independent assays), covering 7 magnitude orders, which were all within the acceptable criteria. The approach enabled an LOD and LOQ down to 0.01% (*w*/*w*) (*n* = 12 from 3 independent assays), corresponding to 0.1 g of *G. biloba* per 1 kg of *C. sinensis*.

Compared with the recent report of Dhivya et al. [21], describing a species-specific real-time PCR with a hydrolysis probe targeting the *matk* gene, the present approach achieved similar performance parameters in terms of PCR efficiency and *R*^2^ using serially diluted leaf DNA of ginkgo. However, the proposed real-time PCR method provides a much wider dynamic range (seven orders of magnitude) and a higher sensitivity (0.02 pg of ginkgo DNA) than that obtained by Dhivya et al. [21] (five orders of magnitude and 10 pg of ginkgo DNA). Regarding specificity, the proposed primers and probe targeting the ITS region do not provide any cross-reactivity with any of the known potential adulterants (*Sophora japonica* and *Fagopyrum esculentum* Moench) (Figure S2), while the method of Dhivya et al. [21] was reactive with *S. japonica* at late amplification cycles, which compromised its sensitivity, and the potential reactivity with *F. esculentum* Moench was not verified by the referred authors. Therefore, the proposed method demonstrated full specificity and high sensitivity for gingko detection, with the important achievement of providing, for the first time, a normalized quantitative real-time PCR approach to enable a determination of the proportion of ginkgo in herbal products.

#### 3.2.2. Method Validation

To proceed with the validation of the method, the precision and accuracy should also be evaluated [25,26]. Therefore, blind mixtures containing 20.0%, 8.0%, 2.0%, and 0.2% (*w*/*w*) of *G. biloba* in *C. sinensis* were used. The results regarding the estimated values (%) of ginkgo and the comparative analysis with the real values are presented in Table 3. The obtained values exhibited adequate coefficients of variation (CV), which were between 5.6–17.9% and, therefore, lower than the maximum acceptable (25%), demonstrating the high precision of the method over the considered dynamic range. Regarding the accuracy, three out of the four blind mixtures presented bias values in the range of 5.6–17.9%, being within the recommended range (±25%) [26]. Although the mixture with 0.2% (*w*/*w*) presented a slightly higher error (−27.4%), this is the lowest tested level, not likely to occur due to adulteration, but rather from contamination. Besides, according to Kang [36], bias within 25–30% have been considered as acceptable in real-time PCR methods for food analysis.

#### 3.2.3. Analysis of Commercial Herbal Infusions

For assessing the applicability of the method, the normalized real-time PCR system was used to analyze and further verify the authenticity and labelling compliance of several commercial herbal products (herbal infusions). The analyzed herbal infusions were all labelled as containing ginkgo, wholly or partially (Table 1). All the samples produced amplifiable DNA extracts, which were positive for the ginkgo-specific PCR assay. The samples of mixed herbal species were further assayed by quantitative real-time PCR to assess their ginkgo content. The quantitative results demonstrated that, out of five samples of herbal mixtures with labelled ginkgo contents, four samples (#4, #10, #11 and #16) declared 15% of ginkgo, but the obtained contents were within 0.01–2.98%. In particular, sample #10 had only trace amounts (0.01%) of gingko, suggesting its complete substitution with other plant(s). Sample #12 declared 30% of ginkgo, but the obtained content was 9.95%. Consequently, the results of samples #4, #11, #12, and #16 suggest the partial substitution of ginkgo with other plant(s). The other two mixed herbal samples (#14, #15) did not provide any quantitative information regarding gingko, having low estimated amounts (<3%), suggesting again its reduced use. Therefore, the results of mixed herbal products strongly suggest the practice of adulterations, probably due to the high market price of *G. biloba* and its increasing demand, with the industries using less quantity than they declared to raise their profits. 

## 4. Conclusions

In the herein presented work, a new molecular marker of the ITS region was identified for the species-specific detection of *G. biloba* by both qualitative PCR and real-time PCR with a TaqMan probe, providing high specificity and sensitivity, down to 0.02 pg of DNA (0.285 genomic DNA copies). For the effective quantification of ginkgo in herbal products, a novel normalized real-time PCR system based on the ΔCq method was successfully developed using reference herbal mixtures. The method exhibited high performance parameters, namely PCR efficiency, coefficient of correlation and covered dynamic range (50–0.01%), achieving a LOD and LOQ of 0.01% (*w*/*w*) of ginkgo in tea plant. The quantitative approach was further validated with blind mixtures, demonstrating accuracy, repeatability, and trueness within the range of 20–2%. The applicability of the PCR approaches was demonstrated using a set of commercial ginkgo-containing herbal infusions (*n* = 20), confirming the presence of ginkgo in all the products. However, the obtained quantitative results regarding the estimated ginkgo content of seven herbal mixture samples suggest adulterations due to reduction or almost elimination of ginkgo. The proposed system was demonstrated to be a powerful and robust tool for control laboratories and regulatory authorities to ensure labelling compliance of ginkgo-containing herbal products. Since it was demonstrated that the developed method has a high specificity and sensitivity, it can potentially be useful for further detecting *G. biloba* in other processed herbal products or foods.

## Figures and Tables

**Table 1:** Results of the application of qualitative PCR and normalized quantitative real-time PCR systems targeting the ITS1 region (primers Gkb2-F/Gkb2-R) for the detection and quantification of *G. biloba* in samples of herbal infusions and a universal gene (primers EG-F/EG-R).

Samples		Label	Qualitative PCR ^a^		Quantitative Real-Time PCR		
			EG-F/EG-R	Gkb2-F/Gkb2-R	Cq ± SD ^b^ EG-F/EG-R	Cq± SD ^b^ Gkb2-F/Gkb2-R	Estimated Ginkgo (%, *w*/*w*, Mean ± SD) ^c^
1	Ginkgo infusion (leaves)	ginkgo	+	+	NA ^d^	NA	NA
2	Ginkgo infusion (leaves)	ginkgo	+	+	NA	NA	NA
3	Ginkgo infusion (leaves)	ginkgo	+	+	NA	NA	NA
4	Herbal infusion (sachets)	15% ginkgo	+	+	17.03 ± 0.05	24.53 ± 0.32	2.98 ± 0.60
5	Ginkgo infusion (sachets)	100% ginkgo	+	+	NA	NA	NA
6	Ginkgo infusion (sachets)	100% ginkgo	+	+	NA	NA	NA
7	Ginkgo infusion (leaves)	ginkgo	+	+	NA	NA	NA
8	Ginkgo bio-infusion (leaves) (Agr. non EU)	100% ginkgo	+	+	NA	NA	NA
9	Ginkgo infusion (leaves) (Agr. non EU)	100% ginkgo	+	+	NA	NA	NA
10	Tisana MC (Plant mixture)	15% ginkgo	+	+/−	17.85 ± 0.49	34.00 ± 1.11	0.01 ± 0.01
11	Tisana TB (Plant mixture)	15% ginkgo	+	+	15.50 ± 0.18	23.50 ± 0.05	1.98 ± 0.06
12	Ginkgo infusion (sachets)	30% ginkgo	+	+	18.46 ± 0.13	24.12 ± 0.12	9.95 ± 0.79
13	Ginkgo infusion (sachets)	100% ginkgo	+	+	NA	NA	NA
14	Herbal infusion (sachets)	ginkgo (% NL ^e^)	+	+	17.19 ± 0.07	24.80 ± 0.13	2.68 ± 0.24
15	Herbal infusion (sachets)	ginkgo (% NL)	+	+	16.71 ± 0.02	24.75 ± 0.02	2.00 ± 0.26
16	Herbal infusion (sachets) (Agr. non EU)	15% ginkgo	+	+	16.33 ± 0.11	26.64 ± 0.35	0.47 ± 0.03
17	Ginkgo infusion (leaves)	ginkgo	+	+	NA	NA	NA
18	Ginkgo infusion (leaves)	ginkgo	+	+	NA	NA	NA
19	Ginkgo infusion (sachets)	100% ginkgo	+	+	NA	NA	NA
20	Ginkgo infusion (leaves)	ginkgo	+	+	NA	NA	NA

^a^ (+) Positive amplification; (−) Negative amplification; +/− Doubtful amplification. ^b^ Mean cycle of quantification (Cq) values ± standard deviation (SD) (*n* = 4). ^c^ Mean percentage (%) values ± SD (*n* = 4). ^d^ NA—not applicable. ^e^ NL—not labelled.

**Table 2:** Data of primers used, targeting the ITS1 region of Ginkgo biloba and a conserved eukaryotic region.

Species	Target	Primer	Sequence (5′→3′)	Length	Reference
*G. biloba*	ITS1	Gkb2-F	GCGGTAAGCCCATCTCTCGA	175 bp	This work
		Gkb2-R	CCGAAGCGAACCCGAACAAC		
		Gkb2-P	FAM-ATGCCAAGGTCGCCGGACCGTC-BHQ1		
Eukaryote	Nuclear 18S rRNA	EG-F	TCGATGGTAGGATAGTGGCCTACT	109 bp	[23]
		EG-R	TGCTGCCTTCCTTGGATGTGGTA		
		EG-P	FAM-ACGGGTGACGGAGAATTAGGGTTCGATTC-BHQ-1		[24]

**Table 3:** Results of the validation assays using the normalized quantitative PCR system applied to blind mixtures of *G. biloba* in *C. sinensis.*

Samples	Ginkgo (%, *w*/*w*)		CV ^b^ (%)	Error ^c^ (%)
	Real Value (%)	Estimated Value ^a^ (%)		
CG–A	20.0	23.58 ± 2.22	9.41	17.9
CG–B	8.0	6.86 ± 0.66	9.55	−14.3
CG–C	2.0	2.11 ± 0.15	7.03	5.6
CG–D	0.2	0.15 ± 0.02	16.88	−27.4

^a^ Mean values ± standard deviation (SD) (*n* = 4) of three independent assays. ^b^ Coefficient of variation (CV). ^c^ Error = ((mean estimated value—real value)/real value) × 100.

## Supplementary Materials

The following are available online at https://www.mdpi.com/2304-8158/9/9/1233/s1, Table S1: Results of cross-reactivity testing of ITS1 primers are presented. Figure S1: Sensitivity by qualitative PCR. Figure S2: Analytical specificity by real-time PCR.
