# Supplementary material for: Authentication of Ginkgo biloba Herbal Products by a Novel Quantitative Real-Time PCR Approach
Source: Foods. 2020 Sep 4;9(9):1233. doi: 10.3390/foods9091233 (PMC7555165; doi:10.3390/foods9091233)
Supplement: Supplementary file 1 [file foods-09-01233-s001.pdf]

Supplementary material

Table S1 – Results of PCR amplification targeting the ITS1 region of ginkgo and a universal eukaryotic DNA region of several relevant plant species for cross-reactivity testing

Common name	Plant species	Source	End-point PCR	
			18S rRNA (EG-F/EG-R)	ITS1 (Gkb2-F/Gkb2-R)
Ginkgo	*Ginkgo biloba*	Botanical Garden of Univ. Porto, Portugal	+	+
Ginkgo	*Ginkgo biloba*	Botanical Garden of Bern, Switzerland	+	+
Ginkgo	*Ginkgo biloba*	Botanical Garden of Madeira, Portugal	+	+
Ginkgo	*Ginkgo biloba*	Serralves Garden, Porto, Portugal	+	+
Japanese pagoda tree	*Sophora japonica*	USDA Grin, Univ. of Arizona Herbarium (Tucson, USA)	+	-
Arizona mescal bean	*Sophora arizonica*	Kew Gardens (West Sussex, UK)	+	-
Buckwheat	*Fagopyrum esculentum* Moench	Commercial	+	-
Fringeleaf necklacepod	*Sophora stenophylla*	Kew Gardens (West Sussex, UK)	+	-
Kōwhai	*Sophora microphylla x godleyi*	Kew Gardens (West Sussex, UK)	+	-
Prince herb	*Cymbopogon citratus*	Commercial	+	-
Fennel	*Foeniculum vulgare*	Commercial	+	-
Dandelion	*Taraxacum officinales*	Commercial	+	-
Field horsetail	*Equisetum arvense*	Commercial	+	-
Lemon balm	*Melissa officinalis*	Viseu, Portugal	+	-
Chamomile	*Matricaria chamomilla*	Commercial	+	-
Tea plant	*Camellia sinensis*	Commercial	+	-
Salvia	*Salvia officinalis*	Commercial	+	-
Mentha piperita	*Mentha x piperita*	Commercial	+	-
Borututu	*Cochlospermum angolense*	Commercial	+	-
Gorse	*Pterospartum tridentatum*	Commercial	+	-
Mallow	*Malva sylvestris*	Commercial	+	-
Alexandrian senna	*Senna alexandrina* Mill	Commercial	+	-
Mate herb	*Ilex paraguariensis*	Commercial	+	-
Linden	*Tilia argentea*	Commercial	+	-
Bearberry	*Arctostaphylos uva-ursi* L.	Commercial	+	-
Herb-Robert	*Geranium robertianum* L.	Commercial	+	-
Artichoke	*Cynara scolymus* L.	Univ. Lisbon, Portugal	+	-
Passiflora	*Passiflora incarnata* L.	Commercial	+	-
Boldo	*Peumus boldus*	Commercial	+	-
Verbena	*Verbena officinalis* L.	Commercial	+	-
Hawthorn	*Crataegus monogyna* Jacq	Commercial	+	-
Valeriana	*Valeriana officinalis* L.	Commercial	+	-
Milk thistle herb	*Silybum marianum* L.	Commercial	+	-
Lemon verbena	*Aloysia citrodora*	Commercial	+	-
St John's wort	*Hypericum perforatum*	Commercial	+	-
Hipericão do Gerês	*Hypericum androsaemum*	Amarante, Portugal	+	-
Eucalyptus	*Eucalyptus obliqua* L'Hér	Coimbra, Portugal	+	-
Turmeric	*Curcuma longa* L.	Commercial	+	-
Ginger	*Zingiber officinale*	Commercial	+	-
Oregano	*Origanum vulgare* L.	Commercial	+	-
Basil	*Ocimum basilicum* L.	Commercial	+	-
Coriander	*Coriandrum sativum* L.	Commercial	+	-
Tomato	*Solanum lycopersicum*	Commercial	+	-
Onion	*Allium cepa*	Commercial	+	-
Parsley	*Petroselinum crispum*	Commercial	+	-
Pepper	*Piper* spp.	Commercial	+	-
Bay Leaf	*Laurus nobilis*	Commercial	+	-

Almond	*Prunus dulcis*	Commercial	+	-
Hazelnut	*Corylus avellana*	Commercial	+	-
Chestnut	*Castanea sativa*	Commercial	+	-
Peanut	*Arachis hypogaea*	Commercial	+	-
Walnut	*Juglans regia* L.	Viseu, Portugal	+	-
Horse chestnut	*Aesculus hippocastanum* L.	Viseu, Portugal	+	-
Pine nut	*Pinus pinea*	Commercial	+	-
Soybean	*Glycine max* L.	Commercial	+	-
Sunflower	*Helianthus annuus*	Commercial	+	-
Pumpkin	*Cucurbita mixta*	Commercial	+	-
Rapeseed	*Brassica napus*	Commercial	+	-
Lupine	*Lupinus albus*	Commercial	+	-
Fava bean	*Vicia faba*	Commercial	+	-
Olive	*Olea europaea* L.	Commercial	+	-
Wheat	*Triticum aestivum*	Commercial	+	-
Barley	*Hordeum vulgare*	Commercial	+	-
Rye	*Secale cereale*	Commercial	+	-
Oat	*Avena sativa*	Commercial	+	-
Rice	*Oryza sativa*	Commercial	+	-
Fig	*Ficus carica* L.	Viseu, Portugal	+	-
Bitter orange	*Citrus aurantium* L.	Commercial	+	-
Cherry	*Prunus avium* L.	Commercial	+	-
Peach	*Prunus persica*	Commercial		
Plum	*Prunus domestica*	Commercial	+	-
Apricot	*Prunus armeniaca*	Commercial	+	-
Vine	*Vitis vinífera* L.	Commercial	+	-
Blackberry	*Morus* spp.	Commercial	+	-
Strawberry	*Fragaria* spp.	Commercial	+	-
Mango	*Mangifera indica* L.	Commercial	+	-
Pineapple	*Ananas comosus*	Commercial	+	-

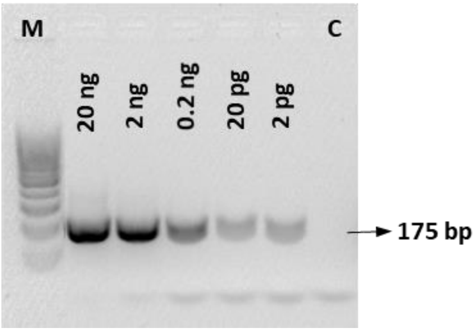

Fig. S1. Agarose gel electrophoresis of PCR fragments of ITS1 region of *Ginkgo biloba* serially diluted DNA extracts. M, 100 bp DNA ladder (Bioron, Ludwigshafen, Germany); C, negative control.

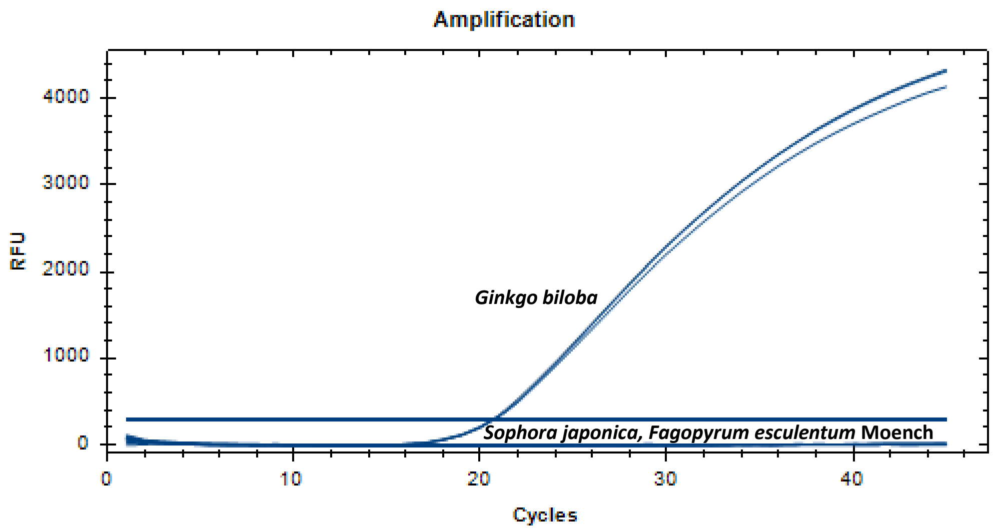

Fig. S2. Amplification curves of a real-time PCR assay with a hydrolysis probe targeting ITS1 region of *G. biloba* to assess the reactivity with *Sophora japonica* and *Fagopyrum esculentum* Moench.
